# Classification and regression trees for epidemiologic research: an air pollution example

**DOI:** 10.1186/1476-069X-13-17

**Published:** 2014-03-13

**Authors:** Katherine Gass, Mitch Klein, Howard H Chang, W Dana Flanders, Matthew J Strickland

**Affiliations:** 1 Department of Epidemiology, Rollins School of Public Health, Emory University, 1518 Clifton Rd, Atlanta, GA 30322, USA; 2 Department of Environmental Health, Rollins School of Public Health, Emory University, 1518 Clifton Rd, Atlanta, GA 30322, USA; 3 Department of Biostatistics and Bioinformatics, Rollins School of Public Health, Emory University, 1518 Clifton Rd, Atlanta, GA 30322, USA

**Keywords:** Air pollution, CART, Classification and regression trees, Multipollutant, Mixtures, Pediatric asthma

## Abstract

**Background:**

Identifying and characterizing how mixtures of exposures are associated with health endpoints is challenging. We demonstrate how classification and regression trees can be used to generate hypotheses regarding joint effects from exposure mixtures.

**Methods:**

We illustrate the approach by investigating the joint effects of CO, NO2, O3, and PM2.5 on emergency department visits for pediatric asthma in Atlanta, Georgia. Pollutant concentrations were categorized as quartiles. Days when all pollutants were in the lowest quartile were held out as the referent group (n = 131) and the remaining 3,879 days were used to estimate the regression tree. Pollutants were parameterized as dichotomous variables representing each ordinal split of the quartiles (e.g. comparing CO quartile 1 vs. CO quartiles 2–4) and considered one at a time in a Poisson case-crossover model with control for confounding. The pollutant-split resulting in the smallest *P-*value was selected as the first split and the dataset was partitioned accordingly. This process repeated for each subset of the data until the *P-*values for the remaining splits were not below a given alpha, resulting in the formation of a “terminal node”. We used the case-crossover model to estimate the adjusted risk ratio for each terminal node compared to the referent group, as well as the likelihood ratio test for the inclusion of the terminal nodes in the final model.

**Results:**

The largest risk ratio corresponded to days when PM2.5 was in the highest quartile and NO2 was in the lowest two quartiles (RR: 1.10, 95% CI: 1.05, 1.16). A simultaneous Wald test for the inclusion of all terminal nodes in the model was significant, with a chi-square statistic of 34.3 (p = 0.001, with 13 degrees of freedom).

**Conclusions:**

Regression trees can be used to hypothesize about joint effects of exposure mixtures and may be particularly useful in the field of air pollution epidemiology for gaining a better understanding of complex multipollutant exposures.

## Background

Every day we breathe a blend of air pollutants, ingest an assortment of nutrients, and are influenced by a unique combination of genes. Throughout the course of a day and lifetime our total exposure can be conceptualized as a complex mixture of different individual exposures. Advances in science have improved our ability to measure these exposures; a major challenge is how best to characterize and relate these mixtures to health endpoints.

Characterization of mixtures for epidemiologic research depends upon both the data that can be obtained as well as the research question of interest. For some research questions interest may center on estimating the combined “joint effects” of two or more individual exposures on a given outcome. Encompassed in this issue of joint effects is the concept of interaction. While some joint effects may be indicative of interaction, it is not always the case. For example, given an additive or multiplicative scale, exposures A and B may combine synergistically, antagonistically, or without interaction to promote disease, and our conceptualization of joint effects encompasses all of these. Here we refer to “interaction” as statistical interaction or effect measure modification, that is a deviation from the expected independent joint effect of two or more risk factors [1].

Statistical interaction is often assessed by including the product of two or more risk factors (exposures) in a regression model and using statistical tests to determine whether the resulting coefficient differs significantly from zero. As the number of exposures increases, the number of possible third-, fourth-, fifth-, and higher-order interactions becomes too large to include in any one model and these are rarely considered in conventional analyses. Testing only a specific sub-set of these interaction terms requires substantial *a priori* knowledge about complex interactions. As model complexity grows so does the challenge of interpretation [2]. In addition, parameter estimates may become unstable as the number of interaction terms increases.

In this paper we describe how classification and regression trees (C&RT) can be used as an alternative method for identifying complex joint effects, including interactions, for multiple exposures. The proposed approach expands the applicability of C&RT to epidemiologic research by demonstrating how it can be used for risk estimation. We view this method as a means to generate hypotheses about joint effects that may merit further investigation. We illustrate this approach with an investigation of the effect of outdoor air pollutant concentrations on emergency department visits for pediatric asthma.

## Methods

### Data

The data we use to demonstrate our C&RT approach are from the Study of Particles and Health in Atlanta (SOPHIA) [3]. The 3-day moving average population-weighted concentrations of ambient carbon monoxide (CO), nitrogen dioxide (NO2), ozone (O3), and particulate matter less than 2.5 microns in diameter (PM2.5) were calculated using measurements from stationary monitors from January 1st, 1999 – December 31st, 2009 [4]. During the same period, daily counts of hospital emergency department (ED) visits for asthma in children 2–18 years old were collected from all hospitals in the area. We defined emergency department visits for asthma as all visits with an International Classification of Disease, 9th edition code for asthma (493.0-493.9) or wheeze (786.07). For a greater description of this dataset see Strickland et al. [5].

### Conceptual example

We illustrate our method assuming the goal is examining the joint health risks of CO, NO2, O3 and PM2.5 on ED visits. To simplify this example and aid comprehension, we have chosen to reduce the set of all possible joint effects by classifying the daily concentrations of each pollutant into quartiles. This simplification yields 4^4^ or 256 different types of days, each of which can be viewed as a unique mixture. To study the association of health with these types of mixtures we could calculate a risk ratio for every type of day, choosing the days when all pollutants are in their lowest quartile as the referent group. This would result in 255 risk ratios.

This approach quickly becomes cumbersome as the number of pollutants (or quantiles) increases. Furthermore, it is unlikely that the joint effects for every pollutant-quantile combination are of interest. Some of these mixtures may never occur due to pollutant covariation, while statistical power will be lacking for rarely occurring mixtures. In addition, as the number of quantiles used to classify the pollutant concentrations increases, the differences in the joint effects between two adjacent quantiles of the same pollutant may be trivial. In this situation statistical efficiency would be improved if similar days were grouped. But how should days be grouped? C&RT methods address this issue by taking all possible joint effects and collapsing them into groups that have similar predicted values for the outcome through a recursive partitioning process.

### Statistical methods

C&RT is a non-parametric regression approach. It represents a supervised form of hierarchical clustering in which the data are sequentially split into dichotomous groups, such that each resulting group contains increasingly similar responses for the outcome [6,7]. The end product of a typical C&RT analysis is a dendogram illustrating the paths of dichotomous splits. Every tree starts with a “root node” that contains the observations from which the tree will be grown. The observations are then partitioned into two “child nodes” based on the value of an independent predictor variable. The resulting child nodes each contain a subset of the original observations. Each child node may be further partitioned, again based on the value of an independent predictor variable. This process continues until a set of partitioning criteria are no longer met, resulting in terminal nodes. Terminal nodes, by definition, cannot have offspring. The collection of terminal nodes forms a complete partition of the observations in the root node.

When the identification of joint effects is of interest, the C&RT approach offers some potential advantages over traditional parametric modeling approaches. C&RT makes no assumption of a monotonic or parametric relationship with the outcome, is able to identify complex interactions among the predictor variables without *a priori* specification of the interaction terms, and can handle datasets where the number of predictors is high relative to the number of observations. C&RT is a supervised learning approach, meaning it creates partitions based on an outcome variable. This is in contrast to unsupervised learning approaches, such as principal components analysis [8], k-means [9], and self-organizing maps [10], which do not consider the outcome.

Although several statistical packages are capable of running C&RT, including the ‘rpart’ and ‘tree’ packages in R and S-plus, CART® by Salford Systems, SYSTAT, and DTREG, they are limited to varying degrees in their applicability to epidemiologic research. In the health sciences, C&RT is most commonly used as a prediction tool [2]; however, for epidemiologic research, we are more often interested in estimating effects than prediction. In the next section we describe a modified C&RT approach that we believe is more appropriate for effect estimation.

### Modified C&RT approach

As a first step, before performing any partitions of the observations, a referent group of days is selected from all study days and held aside; this referent group is not used in tree construction. The purpose of excluding a referent group is to enable statistical comparisons (i.e. risk ratio) between risk associated with days in the terminal nodes and those in the referent group. For our example, we chose as a referent group the days in which all four pollutants were in their lowest quartile. This is analogous to our referent group selection in the conceptual example.

When attempting to estimate causal effects, it is necessary to have a well-specified epidemiologic regression model that controls for confounding. For this example we chose a Poisson generalized linear model using a framework equivalent to the conditional logistic case-crossover model [11], with time trends controlled by matching on weekday, month and year, and meteorology controlled with cubic terms for the three-day moving average: maximum temperature, maximum temperature interacted with an indicator for season, and dew point. A spline for day-of-year with two knots was included to provide additional control for seasonal trends. At this first step the model should not include any of the exposure variables of primary interest (i.e. the pollutant variables). Indicator variables are created representing all possible ways to split days into two groups, using each of the individual exposures in the analysis. The number of indicators needed for each exposure will be one less than the number of distinct levels of the exposure. For example, three indicator variables were created for ozone: one indicator comparing quartiles 1 vs. 2–4, a second comparing quartiles 1 and 2 vs. 3 and 4, and a third comparing quartiles 1–3 vs. 4. This was done for all four pollutants, resulting in 12 indicator variables for the 12 possible splitting points. If one prefers to keep the pollutant variable continuous, indicator variables could be created for every possible comparison. For example if the pollutant contained 80 levels, 79 indicators would be defined. Power may be limited with this approach, due to some joint effects having low representation; however, this is not a limitation of the method but rather a consequence of exploring joint effects that occur infrequently.

Each indicator is then included one at a time in the regression model with control for confounding using all the observations (save for those held out in the referent group). After each run of the model the null hypothesis of independence between the outcome and each of the exposure indicators, conditional on the confounding control, is tested and the *P-*value saved. The *P-*values for all possible exposure indicators from the model runs are compared and the smallest *P-*value below a pre-specified alpha level is selected as the first splitting variable. The observations (excluding the referent group) are then partitioned into two child subsets or nodes, each containing the subset of the original observations according to the indicator variable that produced the optimal split. The process repeats itself for each child node, with the regression model being run separately on the two subsets of data and the best splitting point chosen from among the remaining indicators to further partition the child nodes.

Partitioning stops if a minimum child node size is not met, the null hypothesis cannot be rejected for any of the eligible exposure indicators at a pre-specified alpha level, or no further partitions remain. When any of the stopping criteria are met the node becomes a terminal node. The investigator must specify the significance level (alpha) and minimum node size, though this latter criteria is optional; how conservative the stopping criteria are will partly determine the size of the tree. Consequently there is a trade-off between growing a tree large enough to identify potentially important joint effects and running the risk of over-fitting the tree. In our example we specified a two-sided alpha of 0.15 and a minimum node size of 60 observations. The joint effects for the terminal nodes were calculated by including indicator variables for each terminal node simultaneously in the previously described case-crossover model, with the held out data when all pollutants were in the first quartile as the referent, to get adjusted risk ratios for each terminal node. Analytic code was created in SAS® v9.3 (Statistical Analysis System; North Carolina).

## Results

A total of 4,010 days, out of 4,018, with no missing data on air pollution levels and hospital emergency department visits for pediatric asthma were analyzed. There were 131 days with the concentrations of CO, NO2, O3 and PM2.5 all in their lowest quartiles, which were held aside to serve as the referent group, leaving 3,879 days in the dataset to be partitioned.

**Figure 1:**
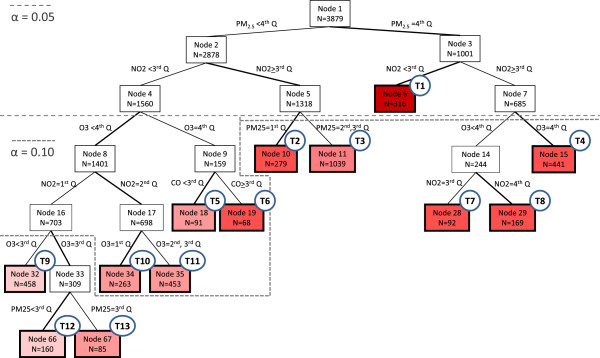
**Tree resulting from C&RT analysis illustrating the joint effects of CO, NO2, O3, and PM2.5, treated as ordinal variables by quartile, for pediatric asthma ED visits in Atlanta from 1/1/1999 – 12/31/2009.** The tree was grown using an alpha of 0.15 and a minimum node size of 60 observations. Nodes are numbered such that each node, *n,* produces two child nodes numbered *2n* and *2n + 1*. Nodes with a bold border are terminal nodes for two-sided α = 0.15, labeled T1 – T13, as indicated by the circle in the upper right-hand corner and are colored according to the strength of association; redder colors indicate a more harmful association. The dotted lines indicate how the tree would appear under different levels of α. For each split of the tree the branch with the more harmful association is bolded.

The C&RT algorithm produced a tree with 13 terminal nodes, based on an alpha of 0.15 (Figure 1). Each terminal node represents a subset of days with a specific pattern of pollutants that the algorithm could not split further, conditional on the confounders included in the model. Referring back to the 256 types of days conceptual example, the terminal nodes will form a partition of the 255 joint effects in the tree. For example, terminal node T1, which represents the subset of days where PM2.5 is in the highest quartile and NO2 is in the 1st or 2nd quartiles, is equivalent to grouping 32 unique types of days – all the combinations of CO and O3, characterized by quartiles, holding PM2.5 constant at the 4th quartile and NO2 at *either* the 1st or 2nd quartile.

The tree depicted in Figure 1 is configured such that the right-hand branch of each split always corresponds to the higher concentration. As a result, the mean concentration of the pollutants in the terminal nodes generally increases from left to right in the tree. Table 1 contains the mean and standard deviations of the pollutant concentrations at each terminal node. The right-most terminal node, T4, has the highest mean concentrations. The referent group contains the lowest concentrations for all four pollutants, by design.

**Table 1:** Mean and standard deviation for pollutant concentrations in each terminal node, Atlanta, Georgia, 1999 - 2009

**Terminal node**	**N**	**CO**	**NO2**	**O3**	**PM2.5**
		**Mean (SD)**	**Mean (SD)**	**Mean (SD)**	**Mean (SD)**
Overall	4010	0.57 (0.3)	21.07 (7)	43.76 (17.45)	14.06 (5.78)
Referent group	131	0.27 (0.04)	11.9 (2.74)	24.68 (3.88)	6.81 (1.4)
T1	316	0.5 (0.18)	16.68 (2.91)	57.09 (14.05)	21.13 (3.76)
T2	279	0.53 (0.21)	24.13 (2.74)	35.77 (9.84)	8.47 (1.05)
T3	1039	0.68 (0.31)	26.05 (4.31)	41.02 (14.89)	13.26 (1.98)
T4	441	0.67 (0.28)	27.3 (5.31)	72.19 (12.72)	23.66 (4.74)
T5	91	0.33 (0.07)	17.03 (2.42)	60.25 (5.05)	13.77 (2.51)
T6	68	0.66 (0.09)	16.54 (3.17)	62.49 (5.81)	13.94 (2.36)
T7	76	0.71 (0.37)	22.63 (1.29)	41.33 (13.27)	19.26 (2.07)
T8	168	1.13 (0.42)	33.31 (7.08)	38.65 (11.11)	21.18 (4.36)
T9	458	0.44 (0.21)	12.69 (2.58)	30.83 (7.76)	9.83 (2.67)
T10	263	0.5 (0.24)	18.46 (1.22)	23.34 (4.87)	10.18 (2.67)
T11	435	0.44 (0.17)	18.27 (1.28)	42.35 (7.06)	11.46 (2.95)
T12	160	0.37 (0.15)	12.74 (2.3)	47.08 (3.42)	10.1 (1.9)
T13	85	0.45 (0.17)	13.65 (1.98)	49.26 (3.99)	14.69 (0.98)

The dotted lines in Figure 1 show how the tree size, and hence the number of terminal nodes, would change if a more conservative alpha of 0.1 or 0.05 were selected. Note that the dotted line for alpha = 0.05 does not mean that the *P-*values for all subsequent splits are greater than 0.05; it only indicates that the *P-*values for the splits occurring at internal (non-terminal) nodes 4, 5 and 7 were greater than 0.05. The *P-*values for the selected splits at each internal node as well as the subset of data to which the splits apply are presented in Table 2. This information can be used to see how the tree size would differ under alternative choices of alpha.

**Table 2:** Quartile contrasts at each internal (Non-Terminal) node

**Internal node no.**^**a**^	**N**	**Quartile contrast**^**b**^	**Wald *P*-value**^**c**^	**Subset of pollutant quartiles to which contrast applies**^**d**^			
				**CO**	**NO2**	**O3**	**PM2.5**
1	3879	PM2.5: 4 vs. 1-3	0.000	All	All	All	All
2	2878	NO2: 3–4 vs. 1-2	0.003	All	All	All	1-3
3	1001	NO2: 3–4 vs. 1-2	0.019	All	All	All	4
4	1560	O3: 4 vs. 1-3	0.096	All	1,2	All	1-3
5	1318	PM2.5: 2–3 vs. 1	0.123	All	3,4	All	1-3
7	685	O3: 4 vs. 1-3	0.128	All	3,4	All	4
8	1401	NO2: 2 vs. 1	0.086	All	1,2	1-3	1-3
9	159	CO: 3–4 vs. 1-2	0.043	All	1,2	4	1-3
14	244	NO2: 4 vs. 3	0.096	All	3,4	1-3	4
16	703	O3: 3 vs. 1-2	0.140	All	1	1-3	1-3
17	698	O3: 2–3 vs. 1	0.062	All	2	1-3	1-3
33	309	PM2.5: 3 vs. 1-2	0.033	All	1	3	1-3

^a^The node numbers correspond to the numbering in Figure 1 (where each node, *n,* produces two child nodes numbered *2n* and *2n + 1).*

^b^Based on the indicator variable chosen for the best split.

^c^*P*-value based on a Wald test that the beta coefficient for the quartile contrast indicator is zero.

^d^Each subset of pollutant concentration levels represents an effect modifier of the quartile contrast and relates directly to the branching of the tree in Figure 1. Note that in the first split of the tree there is no effect modification by any of the pollutants because the entire dataset is used.

A simultaneous Wald test for the inclusion of all 13 terminal nodes in the model was significant, with a chi-square statistic of 34.3 (p = 0.001, with 13 degrees of freedom), a result that was not unexpected, given that the terminal nodes were created through binary splits determined via hypothesis tests. The joint risk associated with days in each terminal node in comparison with risk associated within the held-out referent group are presented as adjusted risk ratios, estimated in a time series analysis using the same case-crossover model and confounding covariates (Table 3). The largest risk ratio was for terminal node T1 (RR: 1.10, 95% CI: 1.05, 1.16) and corresponds to days where concentrations of PM2.5 are in the highest quartile and NO2 are in the lowest two quartiles. Terminal nodes T2 (RR: 1.08, 95% CI: 1.03, 1.14) and T7 (RR: 1.08, 95% CI 1.01, 1.15) had the next largest risk ratios compared to the referent.

**Table 3:** **Risk ratios of emergency department visits for pediatric asthma for days in the terminal nodes as compared to the referent group,**^**a **^**Atlanta, Georgia, 1999–2009**

**Terminal node**^**b**^	**N**^**c**^	**Risk ratio**	**95% Confidence interval**	**Type of days (pollutant quartiles)**			
				**CO**	**NO2**	**O3**	**PM2.5**
Referent	131	1.00		1	1	1	1
T1	316	1.10	(1.05, 1.16)	1-4	1,2	1-4	4
T2	279	1.08	(1.03, 1.14)	1-4	3,4	1-4	1
T3	1039	1.05	(1.01, 1.1)	1-4	3,4	1-4	2,3
T4	441	1.07	(1.02, 1.13)	1-4	3,4	4	4
T5	91	1.03	(0.97, 1.1)	1,2	1,2	4	1-3
T6	68	1.07	(0.98, 1.17)	3,4	1,2	4	1-3
T7	76	1.08	(1.01, 1.15)	1-4	3	1-3	4
T8	168	1.07	(1.01, 1.14)	1-4	4	1-3	4
T9	458	1.01	(0.97, 1.05)	1-4	1	1,2	1-3
T10	263	1.04	(0.99, 1.09)	1-4	2	1	1-3
T11	435	1.03	(0.98, 1.07)	1-4	2	2,3	1-3
T12	160	1.02	(0.97, 1.08)	1-4	1	3	1,2
T13	85	1.04	(0.97, 1.11)	1-4	1	3	3

^a^Days when all pollutants are in the lowest quartile.

^b^Terminal nodes represent different types of days that can be described in terms of the pollutant quartiles.

^c^Each day is in one and only one terminal node; the column sums to 4010.

^d^*P*-values are associated with the null hypothesis that the risk ratio for the pollutant indicator is 1.0.

## Discussion

Many research groups, particularly in genetics, have used recursive partitioning to identify interactions among large numbers of predictor variables [12-14]; however, for the purposes of epidemiologic research we have found the standard C&RT packages to be lacking, to varying degrees. In this paper we present a new C&RT algorithm that is better-suited to epidemiologic research when generating hypotheses about complex joint effects is of interest.

Perhaps the most important way in which the proposed algorithm differs from available C&RT programs is in its control for confounding. Rarely in observational epidemiologic research are we immune to the hazards of confounding. Nonetheless, because most C&RT programs were developed for the purposes of prediction and classification, and not causal inference, they do not directly account for confounding. The typical C&RT approach is to consider all covariates one-at-a-time in the search for the optimal split [7]; however, this one-at-a-time approach ignores confounding. One approach for handling confounding is to first remove the association with the confounders and then fit a regression tree to the residuals [15]; unfortunately, this approach is appropriate only for Gaussian outcomes and cannot be easily applied to the residuals from generalized linear models (e.g. binomial or Poisson data) [16]. Conditional inference trees, first proposed by Hothorn et al. in 2006, offer a framework for recursive partitioning in which the best split is chosen conditional on all possible model splits [17]; however, this approach requires that all covariates in the conditional model be eligible for partitioning. The C&RT algorithm we propose differentiates exposure covariates from control covariates, i.e., it allows for user-defined *a priori* control of confounding while restricting the selection of the optimal splits to the exposure covariates, thereby making this approach better aligned to epidemiologic research when effect estimation is of interest. Bertolet et al. identified many of the same limitations to the existing C&RT approaches and go on to present a similar method for using classification and regression trees that control for confounding with Cox proportional hazards models and survival data [18].

A cited drawback to existing C&RT algorithms is their inability to quantify exposure effect estimates [19]. The C&RT algorithm we have proposed enables effect estimation through the withholding of a common referent group of days during tree construction. This allows for estimation of joint effects across terminal nodes in relation to the pre-specified reference group. Selecting the referent group *a priori* ensures that it does not depend on the analysis (i.e. how the algorithm groups the data); otherwise each analysis might yield a different referent group and hinder comparisons across studies. Additionally, such *a priori* selection allows the researcher to define a meaningful referent group.

C&RT does not provide a single statistic that summarizes all the joint effects, nor is it possible to look at the tree and assess whether the algorithm “worked”; C&RT merely identifies the joint effects present in the data. Therefore, we suggest using C&RT as an intermediary step to generate hypotheses about joint effects that exist in the data in order to inform future analyses and studies. For example, terminal node T4 has higher mean concentrations for all pollutants relative to T1 and yet the RR for T1 is greater than T4 (RR 1.10 vs 1.07). While this difference could be due to the relatively small sample sizes or random error, one hypothesis is that splitting NO2 at its 50th percentile (quartiles 1 & 2 vs. 3 & 4), which resulted in terminal node T1, may represent a particularly harmful type of PM2.5 mixture with regards to pediatric asthma. Alternatively there may be certain meteorological factors that promote this specific pollutant covariation and influence personal exposure levels, such as relative humidity. These hypotheses lead to several researchable questions. For example, do days in T1 appear to be dominated by a single source? Is there evidence that this joint effect is associated with increased risk in other datasets? Does residual confounding or effect measure modification by meteorological factors further explain the relative risks associated with each terminal node?

CO appears only once in the final tree, as a split at internal node 9, which results in terminal nodes 5 and 6 (Figure 1). This suggests that in Atlanta CO may be less associated with pediatric asthma visits than O3, NO2, and PM25. The minimal role of CO in the final tree is not entirely surprising since ambient concentrations of CO in isolation pose no appreciable health risk to the general population [20]; we chose to include CO in our model to act as a potential surrogate for other pollutants emitted from combustion sources that were not included in the model. Removing CO from the analysis – assuming no change to the referent group – would only affect the final tree by collapsing terminal nodes 5 and 6 into a single terminal node.

The RRs in Table 3 do not appear to be dominated by any single pollutant. Instead they suggest that higher levels of pollution are generally more harmful, with the RRs appearing relatively robust to the components of the mixture. Terminal nodes T1, T3, T4, T6, T7 and T8 all have high overall mean concentrations, but from Table 1 it is clear that the distribution of pollutants in these terminal nodes is different. For example, T8 is driven by high NO2, CO and PM25; T4 by high O3 and PM25; and T6 by high O3, and yet all three terminal nodes are associated with a similarly elevated risk relative to the referent group. These results are consistent with a recent multipollutant study by Winquist et al., which found that the joint effects of an inter-quartile range increase pollutant combinations (oxidants, secondary pollutants, indicators of traffic, power plants, and five criteria pollutants) resulted in statistically significant health effects but that the point estimates for the different pollutant combinations were not appreciably different from each other [21]. From the perspective of multipollutant risk assessment, the C&RT approach of classifying day types may offer valuable insight by identifying specific pollution mixtures that are detrimental to health, which could lead to simultaneous regulation of pollutants or identification of harmful sources. In addition, by calculating a single joint effect for each terminal node, this approach helps to avoid over estimation of the RRs that could occur from joint effect calculations based on single pollutant models in which the single pollutant associations may be capturing the effects of correlated pollutants.

The confidence intervals presented in Table 3 should be viewed in the framework of hypothesis generation and not as a tested result. Multiple significance tests were conducted to identify the terminal nodes. Ideally the joint effects for the terminal nodes would be estimated using independent observations; however, because another independent study was not available at the time of analysis, confidence intervals should not be interpreted at their nominal level. Instead, in the spirit of hypothesis generating, the confidence intervals should be used to motivate future analyses, which may lead to substantive results.

Each terminal node can be interpreted as representing a specific mixture or a collection of mixtures that has a similar association with the outcome. Although the path of exposure indicator terms leading to each terminal node in a C&RT tree may indicate interaction, this is not always the case. For example, suppose a tree splits first after the third quartile of PM2.5 and then both branches go on to split between the second and third quartiles of NO2 (similar to the tree in Figure 1). If the risk ratios comparing the higher NO2 terminal nodes with the respective lower NO2 terminal nodes within levels of PM2.5 are the same (a subjective decision) then interaction is not present. In this scenario, the effect of NO2 on the outcome does not depend on the level of PM2.5, and therefore the tree is not suggestive of interaction. The tree in Figure 1, however, does not meet this criterion. Instead we conclude that interaction between NO2 and PM2.5 *is* present in our data because a calculation of the relative risks comparing days in internal nodes 5 vs. 4 and internal nodes 7 vs. 6 suggests a different direction of effect of NO2 at low vs. high levels of PM2.5 (RR: 1.03 and RR: 0.96 respectively).

In our air pollution example, the fact that internal nodes 4 and 5 split on different exposures (O3 and PM2.5, respectively) suggests that there is something different about the association of the pollution mixtures on pediatric asthma visits on moderate PM2.5 days when NO2 is below vs. above its median level. By looking at the C&RT tree we cannot determine whether this is due to some chemical or physiological interaction between PM2.5 and the other pollutants, a difference in the particles that comprise PM2.5 on high days as compared to low or normal days, the covariation of PM2.5 with other pollutants, random error, or some other factor. Instead, we can use the C&RT tree to generate such hypotheses regarding relationships that exist in the data, which can then be investigated in subsequent analyses. For example, an interesting follow-up analysis would be to perform C&RT on just the PM2.5 constituents to identify the components that appear to be driving the health association.

While most C&RT packages utilize measures of node impurity, including the Gini index for classification trees and least squares for regression trees [7], to guide the splitting decisions there are situations in which other criteria may be justifiable. One approach is to base the best split on statistical significance, as was done in this paper and has been favored by others [17,18]. Selecting splits based on the smallest *P-*value (or largest Chi-square statistic) illustrates how recursive partitioning can be used to capture the strongest association present in the data.

The selection of α in the proposed algorithm is analogous to pruning in the traditional C&RT programs, with larger values of α generating larger trees and smaller values generating nested sub-trees. A frequently cited downside of C&RT is the instability of the tree, leading many investigators to favor random forests instead, which is an approach that incorporates information from an ensemble of trees [6]. Although random forests offer a solution to tree stability, because there is no summary tree created, identification of joint effects is difficult. In the example we have presented, tree size and stability will be affected by the cut-points selected to categorize the exposures. Because the purpose of the proposed approach is hypothesis generation, and not prediction or classification, the stability of any individual tree may be of less concern. Once C&RT has been used to identify potentially harmful joint effects, further refinement of these effects, including investigating a dose–response relationship or finding specific cut-point values, can be conducted using other statistical approaches. Furthermore, knowledge of the *P-*values at each splitting point in the tree, including the most significant and several runners-up, may offer a guide for the stability of any given branch.

C&RT is sometimes criticized for displaying a selection bias towards predictor variables with more splits [17,22]. We tried to address this by assessing equal numbers of potential splits (e.g. quartiles) for each predictor. In our example we chose to create quartile indicators to bridge the C&RT results with the conceptual example; however, the proposed algorithm places no restrictions on how the splits are created. One could create finer splitting points (e.g. deciles or centiles) to better approximate the continuous nature of the exposures; however, if statistical significance is used to determine the best split, the aforementioned tendency to select more balanced splits could become more pronounced as the number of potential splits increases. Allowing the exposures to remain continuous is currently infeasible with this modified C&RT algorithm, due to computational challenges posed by the quantity of GLM models needed, and this direction warrants future methodological development. Alternatively, splits could be based on substantive knowledge (e.g., the U.S. National Ambient Air Quality Standards). An advantage of this approach is that it would allow for greater generalizability, as the splitting points would not be data-based.

A particular challenge in mixtures research is how to deal with highly correlated exposures; while not unique to C&RT, it is important to consider how it may influence the regression tree results. If two exposures are highly correlated, and one is causally associated with the outcome while the other is merely a surrogate for the former, the algorithm will not necessarily split on the causal exposure. This is of particular concern if the two exposures have differential measurement error, as the exposure with the least amount of measurement error can have the estimated greatest effect, even if not causal [3]. Pollutant correlation also affects the frequency at which specific mixtures occur. Of the 256 possible day types referred to in the conceptual example, 37 never occurred during the 11-year period, and another 58 occurred less than 0.1% of the time. This happens because the three-day moving averages of O3 and PM25 are strongly correlated (ρ = 0.61), as are CO and NO2 (ρ = 0.59). The regression tree will have limited power to identify whether rarely occurring exposures are harmful. As a result, the terminal nodes in the resulting tree can be considered as *either* indicative of homogeneity of effect *or* as lacking sufficient power to split further.

C&RT is one of many statistical tools that can be used to address the challenge of multipollutant exposures. Among the more frequently cited approaches are single pollutant regression models [23], two-pollutant regression models [23-25], source apportionment [26], clustering [8-10], recursive partitioning [7,27], dimension reduction [28-30], and Bayesian model averaging [31]. Two recent reviews offer a detailed overview of the advantages and disadvantages of these and other approaches for multipollutant research [19,32]. Recursive partitioning approaches, including C&RT, are attractive because unlike traditional regression models they require no distributional assumptions and can easily handle large numbers of predictors. While C&RT is frequently utilized for its ability to identify complex interactions [33-35], we feel that this should be broadened to “complex joint effects”. Such a broadening of scope would not only help to caution against the misinterpretation of interaction in C&RT trees, a problem that others have documented in the literature [6], but it would also expand the utility of C&RT. Identifying joint effects associated with the outcome may be sufficient if one is interested in describing health associations in terms of covarying exposures where interaction may not exist, as in the case with air pollution. C&RT has the additional advantage over other mixture approaches of producing output that is both visually intuitive and informative.

In air pollution epidemiology, while there is currently interest in moving from a single pollutant to a multipollutant framework, the term “multipollutant” is often used broadly and may encompass many different conceptual issues [23,24,36]. When the multipollutant interest involves the joint effects of several pollutants, we feel that C&RT, particularly with the modifications mentioned in this paper, is a very appropriate tool. We suggest that C&RT be used as an intermediary step for identifying and refining potentially harmful multipollutant joint effects for further investigation. A good example of the benefits of incorporating C&RT into the modeling strategy is demonstrated in Sun et al., who show how a two-step multipollutant modeling strategy involving C&RT and dimension reduction techniques can offer substantial improvements on variable selection [19].

For illustrative purposes we have shown how C&RT can be used to address challenges in the field of air pollution; however, there are many other fields in which exposure mixtures are of interest that may benefit from this C&RT approach. As previously mentioned, researchers in genetics have been using C&RT to identify gene-gene joint effects. The proposed C&RT approach would enable these researchers to expand their current approach to include simultaneous control for biological and environmental factors that may confound the gene-gene associations. Other fields that may benefit from C&RT include nutrition, where understanding the joint effects of nutrient mixtures is of interest, and infectious disease research, where advancements in multiplex assays allow scientists to measure an individual’s exposure to many different antibodies.

## Conclusions

With advances in science and technology, high dimensional datasets are increasingly common, leading many researchers to question how best to characterize and analyze these mixtures of exposures. Many issues arise when dealing with mixtures, including exposure covariation, physiological and chemical interaction, joint effects, and novel exposure metrics. Classification and regression trees offer an alternative to traditional regression approaches and may be well-suited for identifying complex patterns of joint effects in the data. While recursive partitioning approaches such as C&RT are not new, they are seldom used in epidemiologic research. We believe that the aforementioned modifications to the C&RT algorithm, namely the differentiation of exposure and control covariates to account for confounding and the withholding of a referent group, can aid researchers interested in generating hypotheses about exposure mixtures.

## Abbreviations

C&RT: Classification and regression trees; CO: Carbon monoxide; ED: Emergency Department; NO2: Nitrogen dioxide; O3: Ozone; PM2.5: Particulate matter (<2.5 μg); RR: Risk ratio.

## Competing interests

The authors declare that there are no financial or non-financial competing interests associated with this research.

## Authors’ contributions

KG conceived of the methodology, designed the algorithm, performed the analysis and drafted the manuscript. MK aided in the conception of the methodology, algorithm development and drafting of the manuscript. HC provided statistical guidance in the design of the algorithm and analysis. WDF provided statistical and conceptual guidance in the design of the algorithm and analysis. MS oversaw the conception of the methodology, designing of the algorithm, analysis and drafting of the manuscript. All authors read and approved the final manuscript.
